# Measurement of the Infection and Integrity of Monkeypox Virus: A New Method Using PMAxx-ddPCR

**DOI:** 10.3390/ijms26031195

**Published:** 2025-01-30

**Authors:** Giuseppe Sberna, Eliana Specchiarello, Cosmina Mija, Fabrizio Carletti, Sara Belladonna, Enrico Girardi, Valentina Mazzotta, Fabrizio Maggi

**Affiliations:** 1 Laboratory of Virology and Biosafety Laboratories, National Institute for Infectious Diseases Lazzaro Spallanzani—IRCCS, 00149 Rome, Italy; 2 Scientific Direction, National Institute for Infectious Diseases Lazzaro Spallanzani—IRCCS, 00149 Rome, Italy; 3 Clinical and Research Department, National Institute for Infectious Diseases Lazzaro Spallanzani—IRCCS, 00149 Rome, Italy; valentina.mazzotta@inmi.it

**Keywords:** monkeypox virus, mpox, propidium monoazide, PMA, PMAxx, digital droplet PCR, ddPCR, virus integrity, virus infectivity

## Abstract

Mpox, caused by the Monkeypox virus (MPV), is a global public health threat. Virus isolation is the gold standard to confirm MPV infection, but this process can face many challenges. As an alternative, a new method was developed in in vitro settings using 50 µM of propidium monoazide (PMAxx, a DNA-binding agent) coupled with digital droplet PCR (ddPCR). Frozen clinical samples analyzed by PMAxx-ddPCR had a median of 0.8 copies/µL, while untreated samples had a median of 29.8 copies/µL. Since a substantial percentage of reduction was observed in these samples (>80%), it was verified whether this reduction could be due to the freezing process. This hypothesis was confirmed both in vitro and using clinical samples. A gradual increase in the mean percentage of reduction was observed after freezing–thawing cycles of MPV-isolate (59.5−81.4%). Moreover, a different percentage of reduction was observed before (68.2%) and after freezing (97.4%) the specimens, suggesting that the freezing process could reduce the number of complete viral particles. Our study shows strong evidence of the usefulness of PMAxx in clinical settings. PMAxx ensures the detection of intact MPV particles, which improves the accuracy of MPV load measurements. This method not only increases the reliability of MPV diagnosis but also overcomes virus isolation limitations.

## 1. Introduction

Two global Monkeypox disease (mpox) outbreaks were reported, one in 2022–2023, and another in 2024. The World Health Organization (WHO) declared them public health emergencies of international concern [1]. The worldwide proliferation of the mpox virus, known for its severe symptoms such as painful rashes and complications including pneumonia, encephalitis, and fatality, as well as its substantial impact on vulnerable populations such as children and immunocompromised individuals, highlights its status as a significant global public health threat. The outbreaks involved two clades of the Monkeypox virus (MPV): clade IIb and clade Ib, which have persisted from 2022 to 2023 and into 2024, respectively [1]. From 1 January 2022, to 31 October 2024, there have been 115,101 laboratory-confirmed cases of mpox reported to the WHO from 126 countries, including 255 deaths [2]. This was the first time cases and transmission chains have been reported simultaneously in both non-endemic and endemic countries across different regions [3]. While close physical contact with lesions on the skin or mucosal surfaces of individuals infected with mpox has been identified as the primary factor for human-to-human transmission, recent studies indicate that sexual activity could also be a significant route of disease transmission [4,5]. Lesions were found on the genitals, perianal, and inguinal areas of infected individuals who tested positive for the presence of MPV DNA, along with a high prevalence of positive results in semen samples from mpox cases, providing further evidence supporting the sexual transmission route [6,7].

For these reasons, to verify the infectivity of MPV and enhance understanding of its transmission routes, it is essential to conduct viral isolation to determine whether the virus can infect and replicate. While cell cultures are essential for virus isolation, such as when performing serological or host–virus interaction studies, several factors, such as suboptimal sensitivity, prolonged storage of samples, or the presence of antibodies against the virus in clinical samples, can hinder the success of this procedure, making it difficult to establish the true viability and infectivity of the virus [8]. Therefore, it is essential to explore different ways to deal with the difficulties of isolating the virus, maintaining the virus’ integrity, and confirming the infectivity of MPV.

Several studies have explored the use of DNA intercalants capable of distinguishing between intact and non-intact viruses as a potential solution [9,10,11]. Among the proposed intercalants, the most widely used is propidium monoazide (PMA), along with its derivatives, such as PMAxx [10]. PMA and PMAxx can penetrate virions with damaged membranes and bind to viral genomes covalently and irreversibly. This binding process prevents amplification by detaching the polymerase when it encounters the intercalant–genome complex. Thus, PCR amplification and detection exclusively captures genomes from intact virions, disregarding free nucleic acids or structurally incomplete viruses [9,10,11]. Depending on the context, amplification is performed using digital droplet PCR (ddPCR), particularly for the detection of bacteria, or conventional quantitative PCR (qPCR), which is primarily used for virus detection. In this study, we first evaluated the efficacy of PMAxx treatment combined with ddPCR in in vitro experiments with an MPV isolate. This method is innovative for MPV, and ddPCR offers numerous advantages over traditional qPCR, including absolute quantification without the need for standard curves, high precision and sensitivity, reduced PCR bias, and an improved signal-to-noise ratio.

Our purpose was to determine if this combined method can effectively discriminate between intact and non-intact viruses. Additionally, we applied the established method to various clinical samples (including skin lesion swabs (SLS), nasopharyngeal swabs (NPS), rectal swabs (RS), urine, semen, bronchoalveolar lavage (BAL), sera, and plasma) to assess its applicability and reliability in real-world scenarios. By doing so, we aim to provide a more accurate assessment of MPV integrity, which is crucial for understanding transmission dynamics and improving public health responses to outbreaks.

## 2. Results

### 2.1. PMAxx Treatment Decreases MPV Load in Both Live and UV-Inactivated Viruses

**Figure 1:**
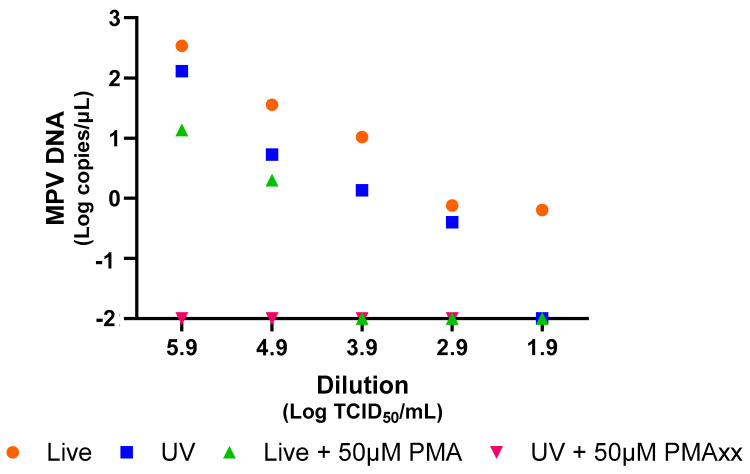
Analysis of live and UV-inactivated MPV by ddPCR, with and without PMAxx treatment at a concentration of 50 µM, using serial dilutions. For graphical representation and statistical analysis, an arbitrary value of −2.0 Log copies/μL was assigned to negative samples.

To verify the efficacy of PMAxx treatment for MPV, serial dilutions of both live and UV-inactivated MPV isolates were treated with 50 µM of PMAxx and analyzed using ddPCR. Figure 1 demonstrates a notable reduction in the copy number of MPV observed in samples treated with PMAxx. This reduction was evident across all serial dilutions for both live and UV-inactivated viruses (Figure 1). Importantly, the application of 50 µM of PMAxx effectively eliminated all damaged viruses within the samples that were previously inactivated using UV light.

**Figure 2:**
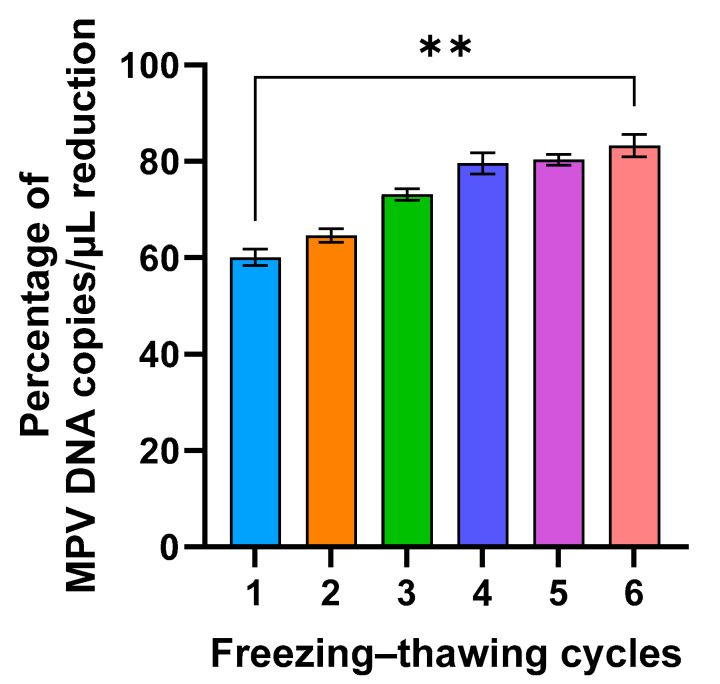
Percentage of MPV copies per µL reduction after six freeze/thaw cycles and 50 µM PMAxx treatment (**: *p* = 0.053).

Furthermore, to investigate if freezing could affect MPV integrity and if PMAxx allowed identification of complete viruses even after thawing, the viral isolate was frozen and thawed six times, and was treated each time with 50 µM of PMAxx. By performing this analysis, it was found that the percentage of reduction increased approximately by 20% between the first and last freezing–thawing cycle, with a statistically significant difference (*p* = 0.0053; Figure 2).

In conclusion, the results of these experiments demonstrate that PMAxx treatment effectively decreases the MPV load in both live and UV-inactivated samples, with this reduction being consistent across all serial dilutions. Furthermore, the repeated freezing and thawing of the viral isolate, followed by PMAxx treatment, further augments the reduction of MPV.

### 2.2. PMAxx Treatment Decreases MPV Load in Frozen Clinical Samples

**Figure 3:**
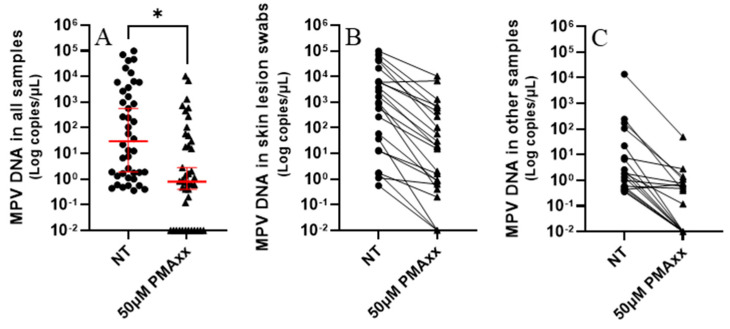
(**A**) Samples not treated (NT) and treated with 50 µM of PMAxx analyzed with ddPCR. Red lines indicate the median and 95% confidence interval. Paired *t*-test (*): *p* < 0.05. (**B**) Skin lesion swabs treated and not treated (NT) with 50 µM of PMAxx analyzed with ddPCR. (**C**) Other samples (8 nasopharyngeal swabs, 5 rectal swabs, 2 sera, 1 plasma, 1 urine, 1 semen, and 1 bronchoalveolar lavage) treated and NT with 50 µM of PMAxx analyzed with ddPCR. For graphical representation and statistical analysis, an arbitrary value of −2.0 Log copies/μL was assigned to negative samples.

Forty-two different frozen clinical samples underwent treatment with 50 µM of PMAxx, followed by analysis using ddPCR. As depicted in Figure 3, a substantial decrease in viral DNA copy numbers was observed post PMAxx treatment. Specifically, samples treated with PMAxx exhibited a median of 0.8 copies/µL, contrasting with untreated samples showing a median of 29.8 copies/µL (Figure 3A). There was a statistically significant difference between the untreated samples and the ones treated with PMAxx (*p* = 0.0193), as shown by the paired *t*-test analysis (Figure 3A). PMAxx treatment reduced the copy number in 97.6% of samples (Figure 3B,C).

**Figure 4:**
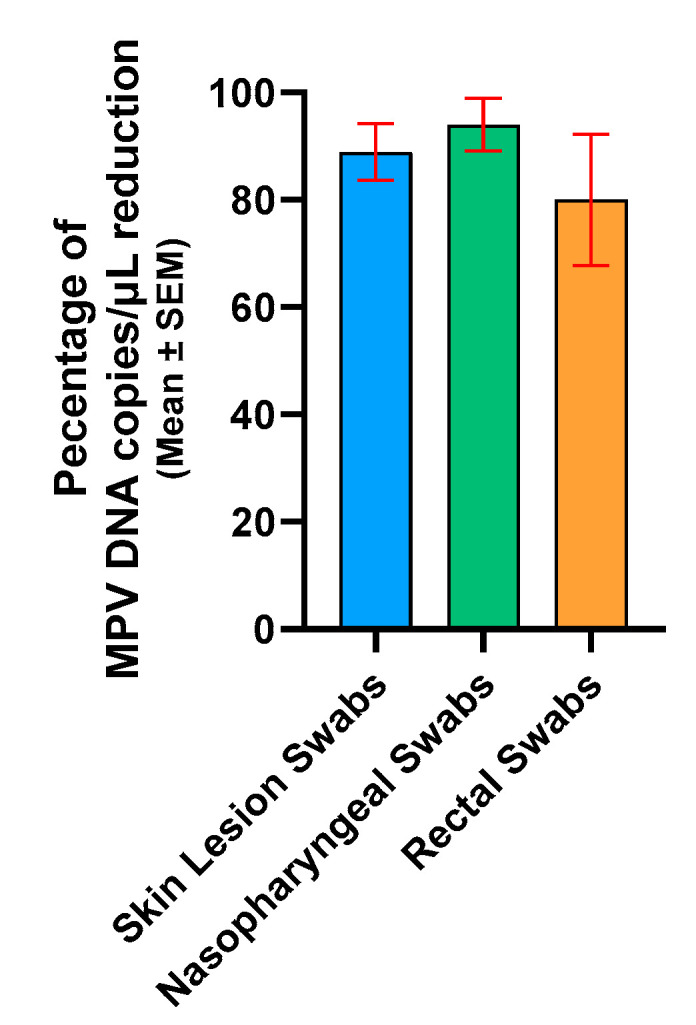
Percentage of reduction in skin lesion swabs, nasopharyngeal swabs, and rectal swabs. For graphical representation and statistical analysis, an arbitrary value of −2.0 Log copies/μL was assigned to negative samples. SEM, standard error of the mean.

Compared by matrix type, the mean percentage of reduction in MPV copies/µL was as follows: SLS had 88.9% (standard error of mean (SEM): ±5.3), NPS had 94.0% (SEM: ±4.9), and RS had 80.0% (SEM: ±12.3) (Figure 4). In conclusion, PMAxx treatment of frozen clinical samples significantly reduced viral DNA, proving effective in detecting non-intact virions across sample types.

### 2.3. Comparison of PMAxx-ddPCR and MPV Isolation Reveals an Association with Viral Copies After Treatment

**Figure 5:**
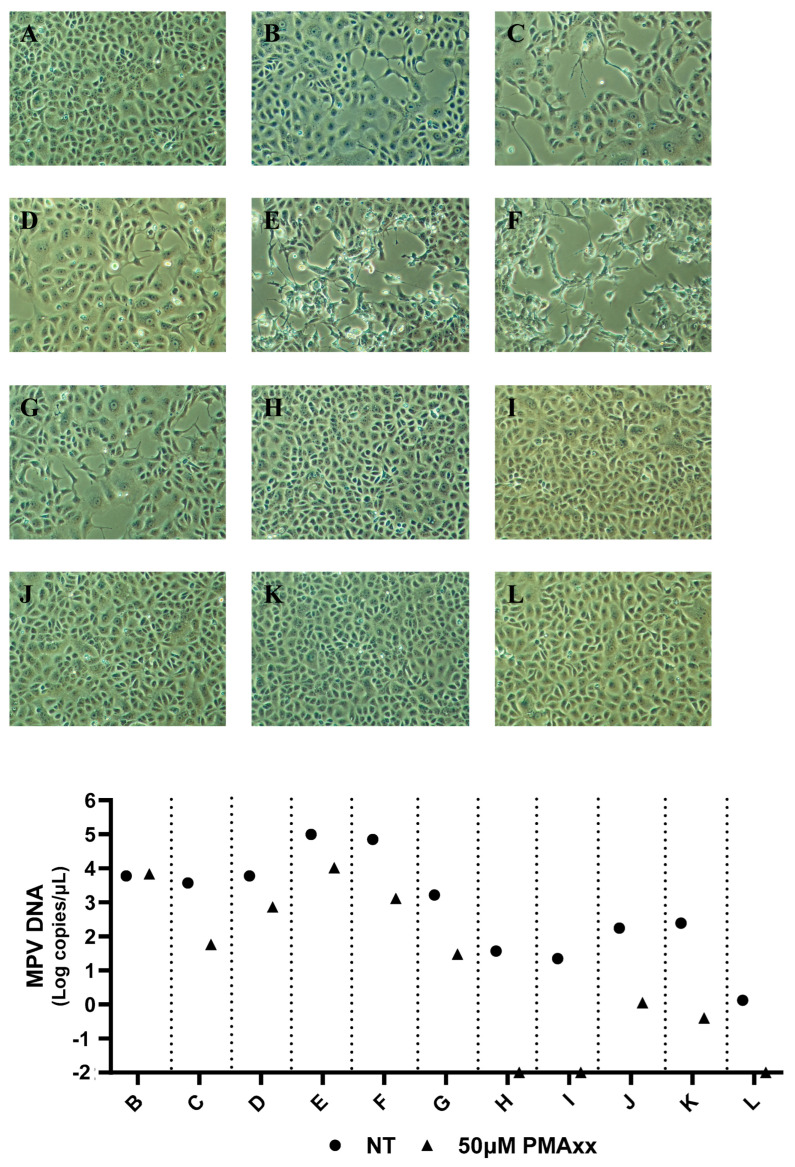
MPV viral isolations of seven skin lesion swabs, two nasopharyngeal swabs, and two rectal swabs and relative MPV DNA Log copies/µL not treated (NT) and treated with 50 µM of PMAxx analyzed with ddPCR. (**A**) Vero E6 cells culture used as a negative control; (**B**–**H**) Vero E6 cells cultured with seven different skin lesion swabs; (**I**,**J**) Vero E6 cells cultured with two different nasopharyngeal swabs; (**K**,**L**) Vero E6 cells cultured with two different rectal swabs; (**A**) Samples not treated (NT) and treated with 50 µM of PMAxx analyzed with ddPCR. Magnification 10×.

To compare the results obtained through PMAxx–ddPCR with MPV isolation (Figure 5), seven SLS, two NPS, and two RS samples were randomly selected. Specifically, the seven SLS samples had a mean of 26,679 MPV copies/µL before treatment (range: 37.2–98,760 MPV copies/µL), while after treatment, a mean of 2770 MPV copies/µL was detected (range: 0–10,396 MPV copies/µL). Only samples with ≥30 copies/µL post-treatment were successfully isolated (Figure 5B–G). The only sample not isolated was the one that had no detectable copies/µL after treatment with PMAxx (Figure 5H). Regarding the two NPS (Figure 5I,J) and the two RS (Figure 5K,L), a mean of 111.3 MPV copies/µL was detected before treatment (range: 1.3–246.4 MPV copies/µL), while after treatment with PMAxx, the number of detected copies/µL dropped below 1.1 (range: 0–1.1 MPV copies/µL). None of these four samples were isolated (Figure 5I–L). In summary, PMAxx treatment significantly reduces the number of detected MPV copies, and only samples with enough post-treatment copies can be successfully isolated.

### 2.4. PMAxx Treatment Reduces MPV Load Less in Fresh than in Frozen Clinical Samples

**Figure 6:**
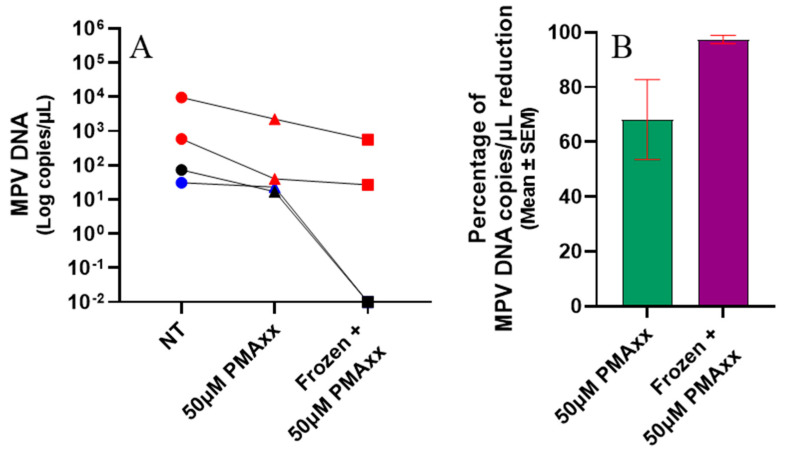
(**A**) Samples were either not treated (NT), treated with 50 µM of PMAxx, or treated with 50 µM of PMAxx after freezing, and then analyzed using ddPCR. The skin lesion swabs are represented by red symbols, the serum samples by blue symbols, and the nasopharyngeal swabs by black symbols. (**B**) The graph shows the percentage reduction in analyzed samples treated before and after freezing. Red lines indicate the mean ± standard error of mean (SEM). For graphical representation and statistical analysis, an arbitrary value of −2.0 Log copies/μL was assigned to negative samples.

Four specimens (1 NPS, 2 SLS, and 1 serum), collected from a single patient, were treated with PMAxx both before and after freezing to verify further how freezing can impact the integrity of the MPV viral particle and, particularly, how PMAxx acts under these two different conditions. As shown in Figure 6, the treatment effect on samples before freezing produced a mean percentage of reduction in MPV copies/µL of 68.2%, while after freezing the mean percentage of reduction increased to 97.4% (Figure 6A,B). The findings indicate that freezing increases the effectiveness of PMAxx treatment, resulting in a greater reduction in MPV load.

## 3. Discussion

Advanced diagnostic techniques, such as PMAxx treatment in conjunction with ddPCR, enhance the accuracy of viral load assessments. This method improves outbreak management by quickly distinguishing between intact and non-intact virus particles, significantly reducing detection time compared to traditional methods, such as virus isolation [11,12,13,14,15,16,17]. PMAxx treatment combined with ddPCR is faster, is more sensitive, and detects only structurally complete viruses. It works with various samples, provides strain-specific information, and offers higher accuracy due to its specificity, precise quantification, and ability to detect low levels of intact viruses. Overall, this method enables the selective quantification of intact viral particles, ensuring that the measured viral load excludes non-infectious viruses. This enhances the specificity and accuracy of the viral load assessment.

Most studies have used in vitro experiments to develop and verify genome intercalants [11,12,13,14,15,16,17]. Additionally, wastewater analyses have identified intact viruses which may be potentially infectious [18,19]. During the SARS-CoV-2 pandemic, methods using PMAxx pre-treatment were developed [11,12], useful for determining if NPS, which test positive for long periods, contain intact viruses [12]. Our study demonstrates the value of this genome intercalant, especially the established method, in detecting whole MPV particles during the recent mpox outbreak, both in vitro and in various diagnostic samples. The method was developed using various virus concentrations and a pre-treatment with 50 µM of PMAxx. While varying concentrations of PMAxx could be required for different samples, a concentration of 50 µM has been commonly used across various studies [10,12], and this concentration was also found to be optimal for the samples in this study. Therefore, analyses were not conducted with other compound concentrations, especially after confirming that 50 µM of PMAxx effectively blocked the detection of any damaged virus inactivated by UV light (Figure 1). Additionally, in clinical samples, there was a significant difference between treated and untreated samples *(p* < 0.05; Figure 3).

The results obtained using ddPCR coupled with 50 μM of PMAxx are consistent with the viral isolation results (Figure 5). These findings also suggest a cut-off point where samples with <30 copies/μL post-treatment can no longer be isolated via cell cultures.

Since a substantial percentage of reduction in MPV copies/µL was observed after treatment with PMAxx in frozen clinical specimens (Figure 4), it was verified whether this reduction could be due to the freezing process disrupting the external viral structures, reducing the number of complete viral particles. Although freeze–thaw cycles are commonly required for sample storage and transport, they can adversely affect the integrity of viral particles. This process can result in the degradation of viral nucleic acids and compromise the structural proteins of viruses, potentially impacting both nucleic acid-based and protein-based assays. This hypothesis was confirmed in in vitro settings by subjecting the viral isolate to a freezing–thawing series and then analyzing it by PMAxx-ddPCR (Figure 2). A gradual increase in the mean percentage of reduction was observed after each freezing–thawing cycle (from 59.5% to 81.4%), proving that the freezing process reduces the number of complete viral particles, i.e., increases the number of free viral genomes that are blocked by PMAxx and not amplified by ddPCR. This result was also confirmed using four samples that were treated with PMAxx both before and after freezing; a higher percentage of reduction was observed in the freeze-treated specimens (97.4%), compared with the not-freeze-treated samples (68.2%; Figure 6).

In conclusion, the ability to rapidly and accurately distinguish between intact and non-intact viral particles is crucial for both clinical diagnostics and public health interventions. This set-up method not only improves the accuracy of viral load measurements but also enhances our understanding of virus viability in different sample types and after freezing. The results indicate that PMAxx treatment and ddPCR together can effectively detect MPV in clinical settings, which can help improve outbreak prevention and patient care methods. This method could be applied to other viral pathogens, providing wide uses in virology research and diagnostics.

## 4. Materials and Methods

### 4.1. MPV Isolate and UV Inactivation

This study used MPV isolate (hMpxv/Italy/un-INMI-Pt2/2022, clade/lineage IIb B.1, GISAID (Munich, Germany) accession ID: EPI_ISL_13251120 [20]; GenBank (Bethesda, MD, USA): ON745215.1 [21]; EVA-G (Marseille, France) Ref-SKU: 008V-05602 [22]) with a titre of 10^5.9^ TCID_50_/mL measured by the Reed and Muench method [23] on VeroE6 cells. The isolate was diluted to 10^1.9^ TCID_50_/mL for the analysis with PMAxx (Biotium, San Francisco, CA, USA [24]).

To verify the PMAxx (Biotium, San Francisco, USA [24]) activity with the inactivated virus, serial dilutions of MPV (10^5.9^ TCID_50_/mL to 10^1.9^ TCID_50_/mL) were inactivated with UV light for 15 min.

### 4.2. Clinical Specimens

From October 2022 to July 2024, various clinical samples (twenty-five saliva swabs, nine nasopharyngeal swabs, five rectal swabs, one urine sample, one semen sample, one bronchoalveolar lavage, three serum samples, and one plasma sample) were collected for diagnostic purposes from 10 male patients hospitalized at the National Institute for Infectious Diseases “Lazzaro Spallanzani” IRCCS in Rome. These samples were analyzed to evaluate PMAxx activity. Patients had a median age of 39.5 years (IQR: 36–54.5 years). When samples arrived at the laboratory, they were routinely tested for MPV presence and frozen at −80 °C; only two SLS, one NPS, and one serum were freshly analyzed with PMAxx.

### 4.3. PMAxx Treatment

PMAxx treatment was carried out on 100 μL of clinical specimens and 100 μL of serial dilutions of MPV isolate (10^5.9^ TCID_50_/mL to 10^1.9^ TCID_50_/mL), using 50 μM of PMAxx™ Dye (Biotium, San Francisco, USA [24]). The PMA-Lite™ 2.0 LED Photolysis Device (Biotium, San Francisco, CA, USA [24]) was used to photoactivate PMAxx for 30 min, according to the manufacturer instructions [24]. Notably, 50 µM was the most used concentration for PMAxx [10,11], and, in our previous study [12], we found that 50 μM PMAxx was optimal, as increasing it to 200 μM did not change the number of copies detected.

### 4.4. MPV DNA Quantification

MPV DNA was extracted from PMAxx-treated and untreated samples by using the QIAamp Viral DNA Mini Kit (Qiagen, Milano, Italia), according to the manufacturer’s instructions [25].

MPV DNA was quantified using the Bio-Rad QX200 AutoDG Digital Droplet PCR system (Bio-Rad, Hercules, CA, USA [26]), as previously described [8,27]. Briefly, 0.9 μM of primers and 0.25 μM of probe, targeting the G2R gene, were added to ddPCR Supermix (Bio-Rad, Hercules, CA, USA [26]). To maintain consistent quantification, DNA from each sample was processed in three separate wells, and the results were combined during analysis. Following the PCR reaction, the droplets were read using a QX100 droplet reader, and the data were analyzed with QuantaSoft software version 1.7.4.0917 (Bio-Rad, Hercules, CA, USA [26]).

### 4.5. MPV Isolation from Clinical Samples

Viral culture was conducted in the BSL-3 facility using Vero E6 cells, following previously established protocols [27,28]. In summary, samples were diluted in MEM containing antibiotics and antimycotics. The mixtures were left at room temperature for 30 min before being inoculated onto Vero E6 cells. After incubating for 1 h at 37 °C with 5% CO_2_, the inoculum was removed and replaced with MEM containing 2% FBS and the antibiotic and antimycotic solution. The cytopathic effect was then examined under a light microscope.

### 4.6. Statistical Analysis

Data management and analyses (median, 95% CI, IQR, mean, SEM, min–max, and *t*-test) were performed using GraphPad Prism version 9.3.1 (GraphPad Software, Boston, MA, USA [29]). A *t*-test was used to evaluate whether the ddPCR results of NT and PMAxx-treated samples were statistically different. For graphical representation and statistical analysis, an arbitrary value of −2.0 Log copies/μL was assigned to negative samples.

## 5. Conclusions

Our study provides compelling evidence of the effectiveness of PMAxx in clinical settings. By ensuring the detection of undamaged MPV particles, PMAxx enhances the precision of MPV load measurements. This methodological advancement not only improves MPV diagnostic accuracy but also allows us to overcome the limitations of viral isolation. This is crucial for advancing our understanding of MPV infections and for developing more effective strategies for their diagnosis and management in clinical practice.

Additionally, a notable benefit of this method is that it removes the need for a BSL-3 facility to analyze the samples. This makes the process more accessible and feasible in various clinical settings. However, it should be noted that there are additional steps required to handle the PMA, such as the need for photoactivating the PMA for 30 min. In summary, while PMAxx provides significant benefits regarding accuracy and practicality, it is important to recognize both its advantages and the extra steps involved. By doing so, we can better understand its overall impact on MPV diagnostics and clinical management.

## Data Availability

The original contributions presented in the study are included in the article; further inquiries can be directed to the corresponding author.
